# Reducing stress, strengthening resilience and self-care in medical students through Mind-Body Medicine (MBM)

**DOI:** 10.3205/zma001731

**Published:** 2025-02-17

**Authors:** Benno Brinkhaus, Barbara Stöckigt, Claudia M. Witt, Miriam Ortiz, Markus Herrmann, Daniela Adam, Peter Vogelsänger

**Affiliations:** 1 Charité – Universitätsmedizin Berlin, Corporate Member of Freie Universität Berlin and Humboldt-Universität zu Berlin, Institute of Social Medicine, Epidemiology and Health Economics, Berlin, Germany; 2 Otto-von-Guericke University Magdeburg, Faculty of Medicine, Institute of General Practice and Family Medicine, Magdeburg, Germany

## Abstract

Stress-related illnesses are on the rise among medical students and doctors in Germany and internationally. Mind-Body Medicine (MBM) methods are increasingly being used to reduce stress. MBM courses are now being offered to students at medical faculties in Germany, including Charité - Universitätsmedizin Berlin and e.g. at the universities of Magdeburg, Witten-Herdecke, Essen and Ulm. The courses offered in Berlin and Magdeburg are presented as examples in this article. In addition, the Charité course was also conducted with employees (doctors and nurses) of a Charité intensive care unit. The student courses at both medical universities were evaluated at the same time. The results of the analyses showed a reduction in perceived stress and an increase in self-efficacy, mindfulness, self-reflection and empathy in 117 Charité students, and an improvement in mindfulness (Freiburg mindfulness inventory (FFA)) and self-compassion (Self Compassion Scale – German Version (SCS-D)) in 69 students from Magdeburg.

In the qualitative focus groups, the students at Charité also reported better abilities to self-regulate stressful experiences, personal growth and new insights into integrative medicine.

The further implementation of MBM courses at German-speaking medical universities appears to make sense. In addition, MBM courses should be networked across different locations in order to coordinate their content and carry out a joint evaluation using standardised measurement instruments on a larger group of participants. In addition, the implementation of randomised controlled studies to investigate the effectiveness of MBM courses would be beneficial.

## Abstract (de)

Stressbedingte Erkrankungen nehmen bei Medizinstudierenden und Ärzt*innen in Deutschland und international zu. Verfahren der Mind-Body-Medizin (MBM) werden zunehmend zur Stressreduktion eingesetzt. Studierenden werden MBM- Kurse zunehmend auch an medizinischen Hochschulen in Deutschland angeboten, unter anderem an der Charité - Universitätsmedizin Berlin und in den Medizinischen Fakultäten der Universität Magdeburg, Witten-Herdecke, Essen und Ulm. Die Kursangebote in Berlin und Magdeburg werden in diesem Artikel exemplarisch vorgestellt. Darüber hinaus wurde der Charité-Kurs auch bei Mitarbeitenden (Ärzt*innen und Pflegekräfte) einer Charité-Intensivstation durchgeführt.

Die Studierenden-Kurse beider medizinischer Hochschulen wurden begleitend evaluiert. Die Ergebnisse der Analysen zeigten bei 117 Charité-Studierenden eine Verringerung des wahrgenommenen Stresses und eine Steigerung von Selbstwirksamkeit, Achtsamkeit, Selbstreflexion und Empathie, bei 69 untersuchten Studierenden aus Magdeburg fand sich eine Verbesserung der Achtsamkeit (FFA) und des Selbstmitgefühls (SCS-D).

In den Fokusgruppen berichteten die Studierenden an der Charité zudem über bessere Fähigkeiten zur Selbstregulierung von Stresserfahrungen, persönliches Wachstum und neue Einsichten in die Integrative Medizin.

Die weitere Implementierung von MBM-Kursen an deutschsprachigen medizinischen Hochschulen erscheint sinnvoll. Darüber hinaus sollte eine standortübergreifende Vernetzung von MBM-Kursen erfolgen, um sich inhaltlich abzustimmen und eine gemeinsame Evaluation mit standardisierten Messinstrumenten an einem größeren Teilnehmendenkollektiv durchzuführen. Zusätzlich wäre die Durchführung von randomisierten kontrollierten Studien zur Untersuchung der Wirksamkeit von MBM-Kursen zielführend.

## 1. Background

Stress-related illnesses such as sleep disorders, fatigue syndrome, burnout and depression are common among medical students and doctors in Germany and internationally [1], [2], [3]. In a US study conducted among medical residents (doctors in further training), 45% to 75% of those examined showed symptoms of burnout [3]; in another study, around 29% of the doctors assessed showed depressive symptoms [4]. The mean prevalence rate of depressive symptoms or depression in medical students was found to be 27% in a systematic review of 183 studies from 43 countries [5]. In addition, burnout stress increases along the course of their studies and at the beginning of further training [5], [6]. This has serious psychological and physical consequences for those affected and their social environment. For example, stressed or even depressed doctors who are less empathetic and lack concentration are six times more likely to make treatment errors than non-depressed colleagues [7].

A decline in the ability to empathise has been observed among students in the clinical phase [8], [9], a phenomenon that has also been described as “The Devil is in the Third Year” [10]. However, it is not only the loss of empathy, but also the health burdens on medical students and young doctors that give cause for concern and action, a topic that has been discussed for more than a decade due to its urgency. The Geneva Declaration, revised in 2017, obliges doctors to take care of their own health, well-being and skills in order to provide medical care of the highest standard. The German medical profession addressed the issue with a focus topic at the German Medical Conference in Münster in 2019 and with a special issue in the German Medical Journal [1]. 

Mind-Body Medicine (MBM) methods are increasingly being used to reduce stress and improve individual resilience. Resilience is the process and result of successfully adapting to difficult or challenging life experiences, particularly through mental, emotional and behavioural flexibility and adaptation to external and internal demands [https://www.apa.org/topics/resilience]. The aim of this article is to describe the development of MBM, as well as the exemplary implementation of course programmes at two medical universities and their positive evaluation results. 

Mind-Body Medicine is an innovative, integrative concept that links the body with the psyche, teaches self-care and can be used both preventively and therapeutically. Multimodal therapy concepts are designed to reduce symptoms and strengthen self-efficacy [https://sfmbm.org].

### 1.1. The development of Mind-Body Medicine (MBM)

Body and mind strengthening practices form a large and diverse group of evidence-based or evidence-informed procedures or techniques that are used by therapists trained in MBM (e.g. doctors, psychotherapists and members of other professional groups) for prevention and therapy. Examples include mindfulness meditation, autogenic training, tai chi and yoga. Mind-body medicine is a relatively young discipline. In the 1970s and 80s [11], therapeutic concepts were developed in behavioural medicine in the USA, some of which correspond to or complement modern order therapy, which is known from naturopathy as a therapeutic element [12]. Some techniques, such as meditation, have their origins in centuries-old traditional healing systems and religions, but have been decontextualised and secularised in the context of MBM.

The commonalities between order therapy and MBM include a holistic view of the human being (bio-psycho-social-spiritual) and the salutogenetic, i.e. primarily resource-orientated, treatment approach [12]. The concept was summarised in the 1990s by the National Institute of Health in Washington D.C. under the term Mind-Body Medicine [12]. MBM methods are often combined and supplemented with exercise and nutrition therapy as well as lifestyle change measures, such as the courses described below. 

The therapeutic practice of mindfulness and meditation can be assigned to various areas of medicine and psychology. These include MBM [13], [14] and, as already mentioned, naturopathic therapy, psychosomatic medicine and psychotherapy, including the methods of the so-called third wave of behavioural therapy. Mindful behaviour, the practice of meditation and the effects and attitudes they convey have been part of human experience for a very long time and have been developed, cultivated and preserved for thousands of years in some cases. In ancient medicine, for example, Hippocrates’ so-called “Diaita doctrine” contains instructions for a healthy lifestyle [12]. The first traces of modern Mind-Body Medicine can be traced back to Berlin in the 1920s and early 1930s [15], [16].

In the early 1970s, systematic research [17], [18] and the development of mindfulness-based programmes [14], [19] began in the USA. Programs such as MBSR (Mindfulness Based Stress Reduction), MBCT (Mindfulness Based Cognitive Therapy), DBT (Dialectical Behavioural Therapy) and ACT (Acceptance and Commitment Therapy) [20], as well as MBRP (Mindfulness Based Relapse Prevention), are now a widespread and increasingly well-researched part of medicine and psychology. They are used in the treatment of chronic stress and chronic pain, depression and anxiety, addictions [21] as well as functional disorders and chronic inflammation of the gastrointestinal tract [22], [23]. Extensive research has now documented the effects of mindfulness-based practice at a central nervous [24] and cellular level (e.g. Bhasin et al. [25]).

The so-called ReSource Project of the Max Planck Institute for Human Cognitive and Brain Sciences in Leipzig can be seen as a milestone in basic research, which now allows the practice of mindfulness and meditation to be scientifically legitimised and is probably the most comprehensive research project to date on the effects observed during meditation practice [26]. Functional and structural differences between the ‘pain empathy network’ and the “compassion network”, which are particularly relevant for social professions and burn-out phenomena, were already discovered in preliminary studies [27], [28]. ReSource training aims to improve mental health and social skills, for example to reduce stress, achieve greater mental clarity, increase life satisfaction and learn to better understand other people [https://www.resource-project.org/en/]. Sufficiently long and regular meditation practice can reduce stress cortisol levels, improve attention and body awareness, compassion and the ability to take perspective [29]. 

## 2. MBM courses offered at the Charité in Berlin and the Medical Faculty in Magdeburg

Students are now offered Mind-Body Medicine courses at several medical universities in Germany, including the Charité – Universitätsmedizin Berlin and the Medical Faculty of the University of Magdeburg, but also e.g. at the universities in Essen, Witten-Herdecke and Ulm; a brief course description can be found in attachment 1 .

The *Mind-Body Medicine/Stress Reduction course* offered at the Institute of Social Medicine, Epidemiology and Health Economics of the *Charité – Universitätsmedizin Berlin* since the winter semester of 2010/2011 follows the Mind-Body Medicine Skills (MBMS) course concept of Georgetown University in Washington [30] and is aimed at medical students and students of other health professions studying at the Charité. This optional course takes place in small groups of around 10 participants. The group meets regularly over the course of a semester on 10 dates for two hours each, under the guidance of two trained and experienced lecturers (doctors, psychologists and representatives of other health professions who have been trained accordingly and have taken part in at least one course), who continuously accompany the entire course and also take part in the exercises and reflections.

Course participants learn about various methods of mind-body medicine, such as autogenic training, mindfulness and concentration meditation, movement meditation, eating meditation and metta meditation. This individual introduction is intended to provide a deeper insight into the topic and offer the opportunity to share experiences. The central element of the course is the exchange and joint reflection of experiences after the joint practice, which usually creates a positive group dynamic at the end of the course and the course participants increasingly share their professional and sometimes private experiences, especially in dealing with stress. To accompany the course, participants are advised to behave in a self-caring and empathetic manner towards their environment and, in addition to carrying out the meditations they have learnt at home, to exercise regularly, eat healthily and – if possible – keep a diary. Healthy behaviour is asked about in every MBM class and/or documented in weekly logs.

The Institute of General Practice and Family Medicine at the *Otto-von-Guericke-University* launched a pilot project in the 2018/19 winter semester with the clinical elective subject* “less stress, more competence”*, which is funded by the Oberberg Foundation Matthias Gottschaldt [31]. The course leaders in Magdeburg are experienced, psychodynamically trained practising psychotherapists, with well-founded additional qualifications in mindfulness-based psychotherapy (led by Marsha Linehan/Martin Bohus), and who have spent several years as quality circle leaders or participants dealing with the topic scientifically and practically in preparation for the elective course and can look back on many years of meditation practice.

As part of the clinical elective subject, which has been offered every semester since 2018, students are supported in learning techniques and cultivating attitudes that are available in the scientifically based field of mindfulness and meditation. Participants learn and practise techniques based primarily on the core exercises of the ReSource Project, but also exercises from the so-called “third-wave methods” of behavioural therapy (DBT, MBSR, MBCT or MBRP), which can support them in their physical and psychological presence, in maintaining empathy and (self-)compassion, in dealing with difficult feelings and in developing a professional observer function in relation to themselves and others. They deepen their knowledge by discussing what they have learnt in the group. The practice and documentation of meditation and mindfulness exercises at home is also of central importance here. Participants analyse scientific publications on meditation and mindfulness, resilience and empathy research as well as student and doctor health. In addition, doctor-patient communication in general and family medicine is presented, practised and reflected upon under the imperative of mindfulness and self-care. This includes case work on successful GP communication, familiarisation with and practice of the NURSE scheme and Paul Ekman’s research. Techniques and competences already taught during the degree course can be further deepened and differentiated as part of subsequent further and continuing medical training. For this purpose, a *staged model – the Magdeburg Mindfulness Modules (MAM)* – was developed, which, in addition to the courses for students (module 1), includes further medical training (module 2) and continuing medical education (module 3) [31]. 

Both courses, the Berlin course and the Magdeburg course, took place exclusively online during the SARS-CoV-2 pandemic and were very well received by students.

Other MBM courses are offered to students at medical universities in Germany, e.g. at the University of Witten-Herdecke and the University of Ulm. The course for students at the Mind Body Medicine Summer School in Essen (led by Prof G. Dobos, Dr A. Paul) [https://www.nhk-fortbildungen.de/16-0-Mind-Body-Medicine-Summer-School-aktive-Fortbildug-fuer-Medizinerinnen-und-Therapeutinnen.html], which has been taking place since 2006 and was held for the 18^th^ time this year, is also very well established. Over 1,000 participants from Germany and other German-speaking countries have already taken part in this summer school.

In the future, it appears necessary to impart knowledge and experience of MBM to doctors as part of their further training and continuing education and to record the scientific evidence in further studies [31]. This has already been the case since 2019 in the staged model of the Magdeburg Mindfulness Modules (MAM) in further medical training (module 2) and continuing education (module 3) as well as workshops at congresses of scientific societies in general practice and psychosomatics (e.g. DEGAM, DGPM). The Charité course has already been successfully conducted once between January and March 2018 for employees (doctors and nurses) of a Charité intensive care unit.

## 3. Evaluation of the course programmes

Since 2012, the course at the Charité in Berlin has been exploratively evaluated as part of an evaluation study without a control group with a pre-post comparison and with additional qualitative focus groups by a person independent of the MBM course. The results of the analyses of quantitative and qualitative data collected from 112 students between 2012 and 2019 indicate numerous positive effects of the course. The quantitative results show a reduction in perceived stress and an increase in self-efficacy, mindfulness, self-reflection and empathy [32]. In the focus groups, students reported better abilities to self-regulate stressful experiences, personal growth and new insights into integrative medicine.

After triangulating the quantitative and qualitative data, it was shown that these effects are anchored in their social context through MBM practice and that an interdependent dynamic is created between the experiences of self and others [32]. As part of a further qualitative approach to analysing the content of a data set, it could be seen that the following five central components, in particular, are addressed and improved in students:

Connectedness with others, self-recognition, stress reduction, improved learning and medical education, so that overall self-care and self-reflection are improved in the sense of health-related self-strengthening [33].

The positive results of the MBM course found in Berlin confirm the previously obtained long-term results [34] of this course format, which also showed effects on stress biomarkers [35]. 

Initial results from the Magdeburg MBM project, which is also being scientifically evaluated with a pre-post comparison of the data, also indicate positive effects in 69 medical students using standardised questionnaires and qualitative interviews. These include a significant improvement in mindfulness (FFA) and self-compassion (SCS-D); a publication of the results is currently in preparation.

## 4. Future and perspective

In 2021, a working group of the authors of this article was formed with the aim of exchanging information, learning from experience, reassessing the evaluation instruments used and optimising the evaluation, not only to support and harmonise the nationwide data collection of various medical faculties, but also in the sense of a future evaluation of jointly obtained data.

For this purpose, a network of all working groups offering MBM courses at German-speaking medical universities is planned. The networking of the working groups should also enable better and more rapid further accompanying research in order to scientifically investigate the findings previously obtained abroad and in Germany in a larger group of students, possibly within the framework of larger randomised studies. If further studies show positive effects of MBM courses on students, these should be offered routinely at medical universities. The activities of the Magdeburg working group within the Society for Medical Education and at DEGAM congresses served to promote the exchange and networking of medical faculties in the German-speaking world.

In addition, the Magdeburg project was presented internationally at a workshop organised at the Faculty of Medicine in Marrakesh in 2024. Maintaining the skills learnt by the students in the MBM courses remains a challenge. For capacity reasons, only one-semester courses are currently offered, with course hours (including self-study) limited to around 20-30 (Berlin) or 56 (Magdeburg) (4 semester hours per week). The long-term promotion of resilience and self-care – but not self-optimisation in the sense of “faster, higher, further” – is the goal of the seminars in the self-image of the teachers and students, whereby it is of great scientific and practical interest to follow whether and how the techniques developed by the students and the attitudes to be established with them can become established practice in everyday life, perhaps as naturally as brushing one’s teeth every day.

Thus far, there have been student initiatives in both Berlin and Magdeburg to continue MBM courses without the lecturers in regular meetings after the end of the course. Another option would be to continue the courses online to consolidate the skills learnt, with initial positive experiences reported from Magdeburg. We believe that a rapid dissemination of mind-body medicine for stress reduction, strengthening resilience and self-care among medical students and doctors beyond the specialisms of general practice, psychosomatic medicine and psychotherapy as well as naturopathy and integrative medicine in medical education and training would be helpful and important, and also feasible. We are working on the basis of a staged model that requires us to start with MBM interventions during medical school so that medical students in later semesters or at the beginning of their postgraduate training have better resilience to face the challenges of everyday life.

## Authors’ ORCIDs

- Benno Brinkhaus: [0000-0001-6290-744X]
- Barbara Stöckigt: [0000-0003-2438-876X]
- Claudia M. Witt: [0000-0002-5440-7805]
- Miriam Ortiz: [0000-0002-0889-7890]
- Markus Herrmann: [0000-0002-1293-6725]
- Daniela Adam: [0000-0002-8928-1472]

## Competing interests

The authors declare that they have no competing interests. 

## Supplementary Material

Course descriptions
