# Supplementary material for: Reducing stress, strengthening resilience and self-care in medical students through Mind-Body Medicine (MBM)
Source: GMS J Med Educ. 2025 Feb 17;42(1):Doc7. doi: 10.3205/zma001731 (PMC12086252; doi:10.3205/zma001731)
Supplement: Course descriptions [file JME-42-7-s-001.pdf]

Attachment 1: Course descriptions

Course description 1: MBM student course at the Institute of Social Medicine, Epidemiology and Health Economics at Charité – Universitätsmedizin Berlin

Overview:
The course offered at the Charité was founded by Prof. A. Haramati/Nancy Harazduk from Georgetown University in Washington, DC. After participation in the course in 2010, the manual was translated by Prof. B. Brinkhaus and Prof. C. Witt. The optional course was launched in the winter semester 2010/2011 at the Institute of Social Medicine, Epidemiology and Health Economics. The course comprises 10 course units of 2 hours each, which are offered by two course instructors with groups of up to ten participating students (medicine and other health professions at the Charité).
Each course unit consists of a short mindfulness meditation at the beginning, a feedback round, a main MBM process and a short mindfulness meditation at the end. The central element of the course is the feedback from all participants on the meditations and training sessions. Students can download the audios for the course topics so that they can practice at home. Scientific literature on the course topics is made available to all students and discussed, with the focus of the course being on the practical implementation of the meditation exercises. Students are encouraged to eat and live healthily and to exercise regularly. The course is evaluated online using standardised questionnaires before the first day of the course and after the last day, and a focus group survey is also conducted on the penultimate day of the course.

Course exercises:

- Introduction, drawings I, opening meditation
- Autogenic training and biofeedback
- Eating meditation and mindfulness meditation
- Guided imagery “special place”
- Guided imagery “inner guide”
- Written dialogue
- Metta meditation or forgiveness meditation
- Walking meditation
- Shaking and dancing, focus group
- Drawings II, course conclusion

Attachment 1 to Brinkhaus B, Stöckigt B, Witt CM, Oritz M, Herrmann M, Adam D, Vogelsänger P. *Reducing stress, strengthening resilience and self-care in medical students through Mind-Body Medicine (MBM)*. GMS J Med Educ. 2025;42(1):Doc7. DOI: 10.3205/zma001731

Course description 2: Elective subject "Less stress, more competence". Self-care, communicative competence and professional medical behaviour – not only in general medicine – offered at Faculty of Medicine, Otto von Guericke University Magdeburg

Overview:

The elective subject is based on the ReSource project (led by T. Singer). It was prepared between 2015 and 2018 as part of several quality circles and started in the 2018/2019 winter semester as a pilot project with 7 medical students in the clinical phase of their studies. The elective subject was held on two weekends in the 2018/2019 and 2019/2020 winter semesters and concluded with an attendance day. In the winter semester 2020/21, it took place exclusively via Zoom; since the winter semester 2021/22, it has been offered as a hybrid with one weekend of attendance, one day of attendance, three afternoons (Wednesdays) via Zoom and a final meeting in attendance.

The questionnaires are completed by the students before the start (T0) and at the final meeting (T1). During the course of the elective subject, weekly logs are kept in which the exercise practice and other mindful activities (eating, exercise, domestic activities) are recorded. All students present a scientific paper on topics such as the neurobiology of mindfulness and meditation, the ReSource project, the health burden on medical students and doctors, empathy, self-care and resilience and present these for discussion. The following exercises are practiced together in the practice room of the University Hospital's occupational health management department and at home, and reflected on repeatedly in the group discussion:

Core exercises (based on the ReSource project)

- Count down breathing meditation
- BodyScan (in several variations)
- Loving kindness meditation
- Affect dyad
- Thought observation meditation

Further exercises:

- Mindful yoga of the hands
- Mindful yoga for the feet
- Mindful walking (indoor & outdoor)
- Surfing the wave
- Mountain meditation
- Yoga walk
- Sound check of the moment/orchestra of the world
- Short quiet meditation

Attachment 1 to Brinkhaus B, Stöckigt B, Witt CM, Oritz M, Herrmann M, Adam D, Vogelsänger P. *Reducing stress, strengthening resilience and self-care in medical students through Mind-Body Medicine (MBM)*. GMS J Med Educ. 2025;42(1):Doc7. DOI: 10.3205/zma001731
